# Economic Accessibility of Orthodontic Care in India: A Cross-Sectional Study

**DOI:** 10.7759/cureus.73174

**Published:** 2024-11-06

**Authors:** Mohit Chaturvedi, Rajiv Ahluwalia, Tina Chugh, Dhruv Yadav, Mayank Gupta, Robin Malik

**Affiliations:** 1 Department of Orthodontics, Santosh Deemed to be University, Ghaziabad, IND

**Keywords:** confident smile, dental practices, economic disparities, oral health, orthodontic treatment

## Abstract

Background

The rising demand for orthodontic services is juxtaposed with concerns regarding economic accessibility for diverse socioeconomic groups.

Aim

This study aims to investigate the economic accessibility of orthodontic care in India through a comprehensive questionnaire-based approach.

Material and methods

The survey involved a random sample of 5,000 patients actively seeking orthodontic treatment, recruited from both dental college clinics and private dental practices. A structured questionnaire was administered to gather data on education, income levels, awareness of treatment costs, and perceived barriers to undergoing orthodontic treatment. Descriptive statistics, such as frequencies and percentages, were utilized to summarize variables.

Results

Forty-six point three-two percent (46.32%) of participants expressed the belief that achieving a healthy smile should be recognized as a fundamental aspect of the Right to Health. Fifty-eight point three-four percent (58.34%) of participants felt orthodontic treatment was expensive. Seventy-seven point three percent (77.3%) felt the need for some assistance in managing the expenses of orthodontic treatment.

Conclusion

Governments’ support plays an important role in the promotion of orthodontic care affordability in India, as well as in the general vision of orthodontic treatment as a part of comprehensive health care. Such efforts will help make a healthy and confident smile possible for all, thus achieving the study’s goal of identifying economic factors that affect orthodontic treatment.

## Introduction

Orthodontic care, aimed at correcting malocclusions and enhancing oral health, is a crucial aspect of comprehensive dental services. Orthodontic treatment, with its transformative potential, stands as a cornerstone in enhancing not just dental alignment but, significantly, the aesthetics of a person's smile [1]. Numerous studies and scholarly works have consistently demonstrated the positive impact of orthodontic interventions on smile aesthetics. Through the correction of dental irregularities and malocclusions, orthodontic treatment not only contributes to functional improvements but also plays a pivotal role in elevating the overall visual appeal of an individual's smile [2,3]. In India, a nation marked by its diverse socio-economic landscape, the accessibility of orthodontic care poses a significant concern. Factors such as awareness levels, socioeconomic status, the availability of specialists, current ethnic trends, and cultural norms play a crucial role in shaping the demand and preferences for orthodontic treatment [4-6].

Improving the economic aspect of healthcare access has been identified as a significant component that determines general oral health and so is the case with orthodontic treatment. As mentioned by Laothong and Cheng, there are numerous constraints that people face when accessing orthodontic care, one of them being financial constraints. Laothong noted that financial issues are highly significant predictors of orthodontic treatment motivation among Taiwanese and Thai patients, particularly the cost of treatment [7]. Chambers and Zitterkopf explained that financial barriers, such as the cost of treatment, prevent people from seeking orthodontic treatment, thus supporting the view that orthodontic treatment is costly and unattainable [8]. Given the economic disparities prevalent in India, policymakers, dentists, and other stakeholders in the health of the public should understand the accessibility of orthodontic treatment.

This paper proposes to evaluate the economic feasibility of orthodontic treatment in India, as well as the effect of the socio-economic status of patients on the availability of orthodontic treatment. We seek to establish factors like cost, awareness, and supply that limit access to care with a focus on the population in the lower income bracket. Therefore, through a cross-sectional survey of 5000 participants drawn from different regions, we seek to identify significant economic inequalities that impact orthodontic treatment. The implications of our results will be used to make recommendations on how to enhance affordability and access to these resources through policy adjustments and assistance from both governmental and non-governmental agencies.

This study seeks to understand patients’ views on the affordability of orthodontic treatment and how these insights can help us shape policy strategies to improve equitable access to dental care in India. For instance, the study aims to find out whether patients think that the Right to Health should consist of access to a good smile as an integral part of good health. It also seeks to classify the different types of financial or structural support that patients regard as necessary to render orthodontic treatment more affordable and accessible. The findings are meant to guide policy interventions to improve oral health outcomes for diverse socioeconomic groups in India.

## Materials and methods

A random sample size of 5000 orthodontic patients was collected from various dental colleges and clinics representing metro, urban, and rural areas across India in bilingual, English, and regional languages, ensuring a geographically diverse and representative sample. The data were collected through a form filled out by respondents. Informed consent was obtained from all participants, with parental or guardian consent for those unable to consent independently. Confidentiality was maintained, and participant identities were protected. The sample size was calculated using a confidence level of 95% (1.96 at a 5% level of significance), an estimated prevalence of malocclusion in the Indian population at 85%, and an absolute error of 1% [9]. The research protocol was approved by the ethical review board of Santosh deemed to be University [F.No. SU/2021/092(19)].

This cross-section observational study was planned to define the extent and distribution of malocclusion and other orthodontic problems among patients visiting dental clinics in India. This study was conducted over a period of six months at Santosh Dental College and Hospital, Ghaziabad, India covering the metro, urban, and rural areas to obtain a representative sample. The study used a sampling strategy that aimed to include various geographical areas and all the groups of demographic stakeholders. This approach was meant to increase the external validity of the findings. To select a sample, a multistage sampling method was used, where dental colleges and clinics were first divided into metro, urban, and rural areas and then simple random sampling was used among the colleges and clinics of each stratum.

A structured questionnaire was distributed to 5000 orthodontic patients during their visits to selected dental colleges and private clinics in India, covering metro, urban, and rural areas. The structured questionnaire comprised sections on demographics, financial perceptions of orthodontic treatment as well as awareness of orthodontic treatment. The questionnaire was given to patients by trained data collectors at each location who assisted patients in meeting the inclusion criteria. The questionnaire was either completed independently by the patients or with the help of the data collectors if needed. To ensure uniform data collection, all data collectors followed a standardized procedure. The completed questionnaires were reviewed on-site for accuracy and completeness before submission.

The questionnaire used binary scoring (Yes = 1, No = 0) for yes/no questions and weighted scoring for multiple-choice questions, allowing for composite scores to reflect each participant's awareness, affordability perception, and accessibility views on orthodontic care. The questionnaire comprised three sections: Demographic Data (age, education, occupation, income, family structure), Knowledge and Awareness of Orthodontic Treatment (understanding of orthodontic treatment, the importance of a healthy smile, and familiarity with treatment costs), and Perception of Economic Accessibility and Right to Health (perceptions of affordability, availability of financial support, and views on including orthodontic care in the Right to Health).

Reliability was confirmed with a Cronbach’s alpha of 0.81, indicating strong internal consistency. Construct validity was supported by factor analysis, which showed appropriate clustering of items around awareness, affordability, and access, explaining 72% of the variance. Content validity was ensured by expert reviews. A pilot test with 100 participants improved clarity and response options, with feedback leading to minor revisions; pilot responses were excluded from the main study to maintain data integrity.

Inclusion criteria

The following people were included in the study: patients providing informed consent for participation in the study, individuals meeting survey-based criteria for orthodontic issues (bilingual, English and regional languages), patients aged between 6 and 35 years, willingness to undergo orthodontic treatment, the inclusion of both males and females, patients diagnosed with orthodontic problems, patients with no prior orthodontic treatment to reflect primary orthodontic issues, and patients in generally good health.

Exclusion criteria

The following people were excluded from the study: patients who declined to participate in the survey, individuals outside the age range of 6 and 35 years, pregnant women due to potential risks from diagnostic procedures, patients with systemic conditions or diseases affecting orthodontic treatment, patients currently undergoing orthodontic treatment, history of severe dental trauma or maxillofacial surgery, patients with psychological or cognitive impairments affecting participation, and inability to attend follow-up appointments for data collection.

**Figure 1:**
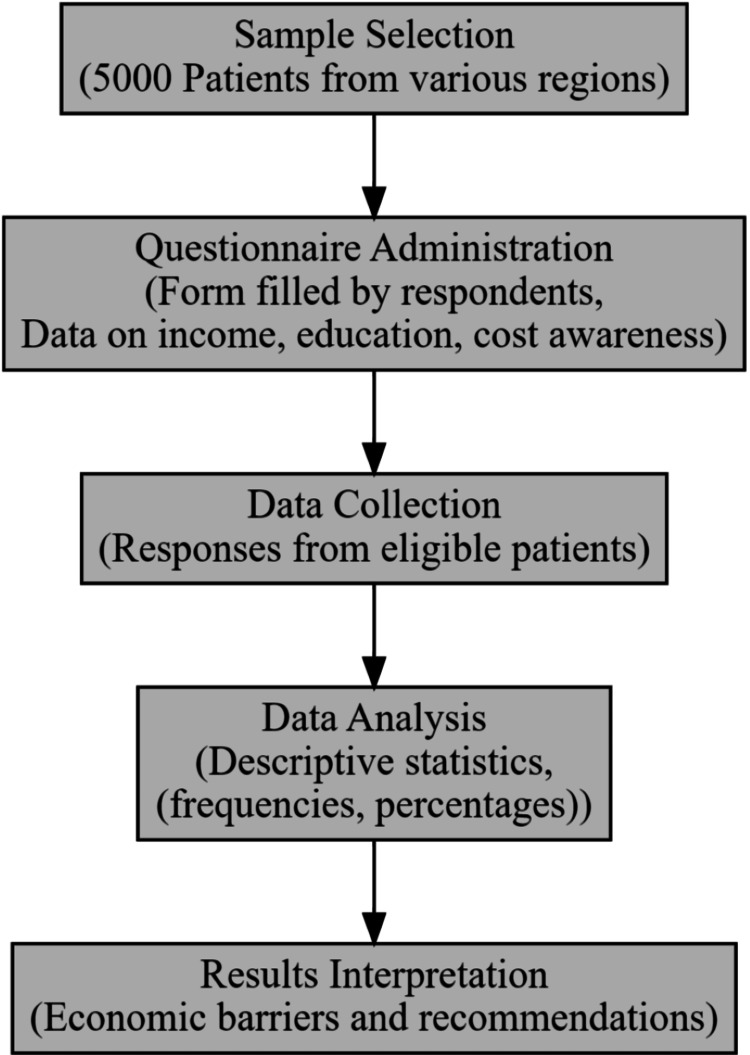
Methodology of economic accessibility study for orthodontic care in India

The flowchart in Figure 1 shows patient selection, data collection, analysis, and the economic barrier interpretation.

Statistical analysis

The data was entered into a Microsoft Excel sheet (Microsoft Corporation, Redmond, WA, US) and further checked for invalid observations, inconsistencies, and any missing observations and outliers.

The statistical tools used in this study were descriptive statistics, which involved the determination of the frequency and percentage of the different data variables. Furthermore, frequency analysis was employed to describe the collected data in terms of frequency distribution and percentage distribution, which are simple descriptive analysis tools. These methods allowed the assessment of the economic feasibility of orthodontic treatment in India in general.

## Results

The age distribution of participants has also been presented in Table 1, the majority (38.88%, 1,944 participants) of the participants are over 28 years, 25-28 years (25.46%, 1,273 participants), 18-24 years (21.04%, 1,052 participants) and 6-17 years (14.62%, 731 participants). This shows that many orthodontic care seeker patients are adults, and more frequent in 28 years age group, which suggests that it could be due to better perception or need for treatment in the older population.

**Table 1:** Distribution of age groups

Age	Frequency	Percentage
6-17 years	731	14.62
18-24 years	1052	21.04
25-28 years	1273	25.46
Above 28 years	1944	38.88
Total	5000	100

Table 2 shows that out of the total respondents, 63.04% of participants had education below graduation. This implies that individuals with lower education levels may have limited knowledge of orthodontic treatments and therefore may not seek this treatment. The highest percentage of participants came from self-employed families (43.02%), followed by private sector employees (32.32%), unemployed (14.36%), and government sector employees (10.3%). A significant portion of participants had a family income of Rs. 10,000 to Rs. 20,000 per month (50.3%) (Table 3).

**Table 2:** Distribution of education

Education	Frequency	Percentage
Below Graduation	3152	63.04
Above Graduation	1848	36.96
Total	5000	100

**Table 3:** Distribution of family’s occupation and family income

Family’s occupation	Frequency	Percentage
Unemployed	718	14.36
Self-employed	2151	43.02
Private sector	1616	32.32
Government sector	515	10.3
Total	5000	100
Family income		
< Rs. 10000 per month	819	16.38
Rs. 10000-Rs.20000 per month	2515	50.3
>Rs. 20000 per month	1666	33.32
Total	5000	100

Table 4 shows that 63.22% of respondents emphasized a good smile as an important factor while 78% did not. Regarding the awareness of orthodontic treatment, 75.02% said they had heard of it and 24.98% did not. This indicates that a good number of people embrace beauty and have some form of knowledge concerning orthodontic treatment. A significant number of participants (59.62%) reported having a family member or friend who had undergone orthodontic treatment. Among those with family/friend experience, 57.52% reported that the orthodontic treatment was completed. Seventy-two percent (72%) of these participants felt orthodontic treatment improved the smile of their family/friends (Table 5).

**Table 4:** Do you feel having a good smile is important and do you have any idea about orthodontic treatment?

How important is a good smile?	Frequency	Percentage
Important	3161	63.22
Not Important	1839	36.78
Total	5000	100
Do you know about orthodontic treatment?		
Yes	3751	75.02
No	1249	24.98
Total	5000	100

**Table 5:** Has any of your family members/friends gone through orthodontic treatment and is their treatment completed?

Have any of your family members gone through orthodontic treatment	Frequency	Percentage
Yes	2981	59.62
No	2019	40.38
Total	5000	100
Was the orthodontic treatment completed?		
Yes	1715	57.5
No	1266	42.5
Total	2981	100
If yes, do you know about orthodontic treatment?		
Yes	1234	72
No	481	28
Total	1715	100

The survey results in Table 6 indicate that the majority of the respondents (58.34%) believe that orthodontic treatment is expensive and 41.66% did not. This means that more than half of the participants consider the cost of orthodontic care as a financial burden. A substantial number expressed interest in improving their smile through orthodontic treatment (78.82%), with 58.34% considering the treatment to be expensive. Among those not interested, cost factor (34.56%), time factor (22.99%), and distance (33.42%) were major reasons (Table 7).

**Table 6:** Do you feel orthodontic treatment is expensive?

Do you feel orthodontic treatment is expensive	Frequency	Percentage
Yes	2917	58.34
No	2083	41.66
Total	5000	100

**Table 7:** Are you interested in improving your smile through orthodontic treatment?

Are you interested in improving your smile through orthodontic treatment?	Frequency	Percentage
Yes	3941	78.82
No	1059	21.18
Total	5000	100
If not, what is the reason?		
Cost factor	729	34.56
Time factor	485	22.99
Distance	705	33.42
Any other	190	9.00
Total	2109	100

Table 8 reveals that the majority of participants (63; 18%) agreed that the “Right to a Good Smile” is applicable in India while 36. 82% disagree. When asked whether the government should regard this right as necessary, 46.32% of respondents agreed, 29.56% disagreed, and 24.12% had other opinions. This is a clear indication that the participants had a general understanding of the importance of the right with nearly half of the participants supporting its recognition by the government as fundamental. Participants recommended that the government should provide more dental facilities (40.42%), non-governmental organizations (NGOs) should take responsibility (36.94%), and payments for treatment should be allowed in installments (18.42%) (Table 9).

**Table 8:** Do you think the Right to a Good Smile is relevant in India, and should it be considered essential by the government?

Do you think Right to a Good Smile is relevant in India?	Frequency	Percentage
Yes	3159	63.18
No	1841	36.82
Total	5000	100
Do you think the Right to a Good Smile should be considered essential by the government?		
Yes	2316	46.32
No	1478	29.56
Others	1206	24.12
Total	5000	100

**Table 9:** What recommendations do you have for managing expenses, and what steps should be considered?

Orthodontic treatment can be costly. What recommendations do you have for managing expenses, and what steps should be considered?	Frequency	Percentage
The government should provide more dental facilities	2021	40.42
Non-governmental organizations (NGOs) should come forward to take responsibility	1847	36.94
Payment for treatment should be given in installments	921	18.42
Others	211	4.22
Total	5000	100

**Figure 2:**
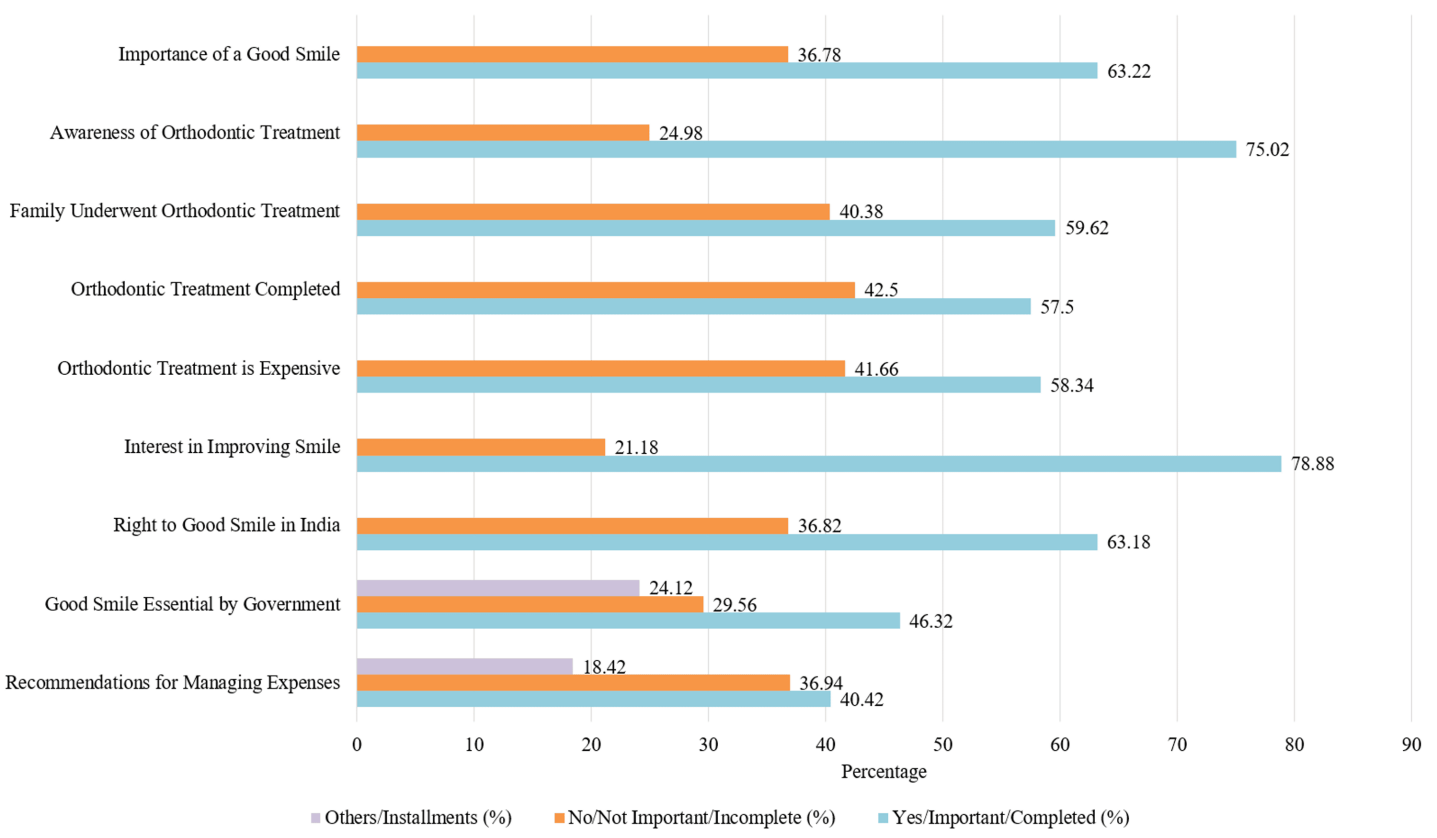
Survey results on orthodontic awareness, treatment accessibility, and perceptions in India

The survey responses depicted in Figure 2 show the perception of the importance of a smile, awareness of orthodontic care, perceived financial barriers, family influence, and perception of government support to underscore the need for intervention to enhance access to orthodontic services.

The questionnaire’s internal consistency was confirmed with a Cronbach’s alpha of 0.81, indicating strong reliability. Factor analysis validated construct validity, grouping items into awareness, affordability, and access domains, collectively explaining 72% of the variance. Composite scores for each domain were calculated, with a range of 0 to 1. The mean awareness score was 0.75 (SD = 0.15), reflecting moderate knowledge of orthodontic treatment. The mean affordability perception score was 0.58 (SD = 0.20), suggesting financial concerns among participants. The accessibility score, representing views on including orthodontic care in the Right to Health, was 0.63 (SD = 0.18), indicating general support for this concept.

A pilot test with 100 participants led to minor revisions for clarity, enhancing the questionnaire’s suitability for the main study. Pilot test responses were excluded from the final analysis to ensure consistency with data collected after the final questionnaire version. Results were influenced by demographic factors: younger participants (18-24 years) and those with education beyond graduation reported higher awareness (mean = 0.78) and a more favorable view of accessibility.

## Discussion

The study was designed to assess the perception of patients regarding the economic accessibility of orthodontic care. The distribution of age groups reflects a diverse demographic seeking orthodontic treatment, with a notable concentration in the age group of 25-28 years. This corresponds with the trend observed in other studies, indicating a higher demand for orthodontic interventions among young adults [10]. Similarly, the predominance of individuals with education levels below graduation underscores the need to address orthodontic accessibility across varying educational backgrounds.

The findings of this study provide valuable insights into the economic and perceptual aspects of orthodontic care in India. The interest in improving smiles (78.82%) aligns with a global trend emphasizing the growing importance of aesthetics in orthodontic considerations [2]. The results of the present study highlight a widespread recognition of the importance of a good smile, with a majority considering it relevant to the Right to Health. The belief that a good smile is essential to the Right to Health is aligned with the broader discourse on oral health and the correction of malocclusion as an integral component of overall well-being [11,12]. However, a substantial portion perceives orthodontic treatment as expensive, indicating a significant economic barrier. The interest in improving smiles is high but concerns about costs and other factors, such as long treatment duration and distance, lead to hesitancy. The reported reasons for not considering orthodontic treatment, such as cost, time, and distance, resonate with a multifaceted array of barriers identified by several studies [7,10,11].

The observed concerns regarding the economic accessibility of orthodontic treatment align with existing literature highlighting the financial barriers that individuals face in seeking dental care, including orthodontics [8,9,13].

The social component of orthodontic treatment is alive and well in the form of socio-economic factors that influence the perception of patients and their families. In India, cultural and financial constraints directly affect decision-making in health care, and therefore, people’s perceptions about orthodontic treatment are shaped by family and social factors. For instance, in families with a member who has successfully completed orthodontic treatment, other family members may also be interested in undergoing the treatment, even if it involves some costs. These influences are particularly felt in areas where health care is inaccessible, and the decision to seek orthodontic treatment is considered as an option of the privileged. Socio-economic factors are also involved since patient’s experiences and perceptions of orthodontic treatment and its benefits may be different between urban and rural or between high and low-economic-status families. Such differences show the need to consider both financial and cultural issues in the formulation of policies for orthodontic treatment in India to increase accessibility to this service in various population groups [14,15].

The belief in the relevance of the Right to a Good Smile in India (63.18%) aligns with a global acknowledgment of the impact of oral health on quality of life and psychosocial well-being [16]. The recommendation for governmental support and the consideration of a good smile as an essential right corresponds with global calls for policy interventions to address oral health disparities [17-19].

The study underscores the economic challenges surrounding orthodontic care, with a significant portion of participants considering it expensive. The expressed desire for governmental assistance signals the urgent need for policy interventions to enhance the affordability and accessibility of orthodontic treatment.

The study highlights the complex interplay of economic factors, perceptions, and sociodemographic influences on orthodontic treatment decisions in India. The identified barriers and recommendations underscore the need for a comprehensive, inclusive, and culturally sensitive approach to enhance orthodontic accessibility. Drawing parallels with global literature emphasizes the universality of challenges and the importance of collaborative efforts in achieving equitable access to orthodontic care.

Limitations of the study

The study may be limited by potential selection bias, as it relies on a specific sample of individuals seeking orthodontic care, potentially excluding those with limited access or awareness. The findings might not be generalizable to the entire population due to regional variations in economic conditions and healthcare infrastructure across India. Furthermore, the cross-sectional study design also brings about response bias while the sample used within the study may not represent the dental workers in rural or deprived areas since the study sample was from dental college clinics and private practices. The study also has limitations in that it does not consider other non-economic factors that may hinder access to orthodontic care, which include time and distance to clinics. These factors suggest that there is a need to conduct more studies to identify other factors that may hinder orthodontic treatment in India.

## Conclusions

The perceived importance of a healthy smile is among the influential aspects that require even more improved action by policymakers. Therefore, governments’ efforts linked to the enhancement of the availability of orthodontic services should target the economic factors that make it difficult for a large number of individuals to gain access to these essential services. With the option that makes it possible for the integration of orthodontics into an overall health system, it will be easier for policymakers to popularize the vision of dentistry as an essential component of human health. Such efforts would not only enhance their health standards but also their self-esteem and networking to form a good society. The opportunity to have orthodontic services expand would go hand in hand with the idea of equal health care opportunity; and irrespective of where the child or the patient is, he/she is equally given the Right to have a Healthy and Attractive Smile. This approach is essential in the progress of society where the Right to Oral Health is well-embraced as an aspect of human rights and human dignity. The study suggests the need for governmental initiatives to enhance accessibility, address cost concerns, and consider the Right to a Good Smile as an essential healthcare component. The recommendations underscore the importance of collaborative efforts involving the government and non-governmental organizations to make orthodontic treatment more accessible and affordable for all.
